# Evaluating Energy Efficiency in Biohydrogen Production and Sustainability Assessment

**DOI:** 10.1021/acsomega.6c03520

**Published:** 2026-09-10

**Authors:** Larissa Castro Ampese, Henrique Di Domenico Ziero, Julián M. Nannini, Leonardo de Freitas Marinho, Gilberto Martins, Tânia Forster-Carneiro

**Affiliations:** † School of Food Engineering, 130229 University of Campinas (UNICAMP), Rua Monteiro Lobato, n.80, Campinas, São Paulo 13083-862, Brazil; ‡ Faculty of Biochemical and Pharmaceutical Sciences, National University of Rosario, Suipacha 531, Rosario S2002LRK, Argentina; § Center for Engineering, Modeling and Applied Social Sciences, 74362 Federal University of ABC (UFABC), Avenida dos Estados, n. 5001, Santo André, São Paulo 09210-580, Brazil; ∥ Center for Information Technology Renato Archer, Rua Dom Pedro I, Campinas, São Paulo 13069-901, Brazil

## Abstract

Fruit by-products are suitable for valorization by different routes. This study aims to valorize apple pomace (AP) and cashew pomace (CP) through a two-stage anaerobic process combining hydrolysis and dark fermentation. In addition, the biogas yield and composition are evaluated, and the sustainability of the process is assessed using green metrics and compared to the literature. A first-order estimate of the energy recoverable from the hydrogen produced is also provided. Two anaerobic reactors in series produced hydrogen using apple pomace (AP) and cashew pomace (CP) as substrates. The total volume of biogas produced in the hydrolysis reactor was 0.065 m^3^, and in the dark fermentation reactor was 0.041 m^3^, resulting in a cumulative hydrogen production of 0.043 m^3^. The study presents a carbonized apple pomace purification cartridge as a preliminary concept. The experimental hydrogen yield was 93.40 m^3^ H_2_ per ton of biomass, representing 40.82% of the H_2_ in the integrated system. In terms of energy, if the hydrogen produced from 1 ton of by-products were used in a fuel cell with 50% efficiency, it would be possible to generate 137.24 kWh of electricity. This study used sustainability analyses (Eco-Scale, Path2Green, AGREE, and GAPI) to evaluate the proposed process. Initial evaluation via green metrics indicates potential sustainability, safety, and efficiency; however, these preliminary findings remain context-dependent. Limitations of the study include mechanistic interpretation based only on indirect indicators, lack of quantitative validation of the purification cartridge, and preliminary, context-dependent energy and sustainability assessments.

## Graphical abstract

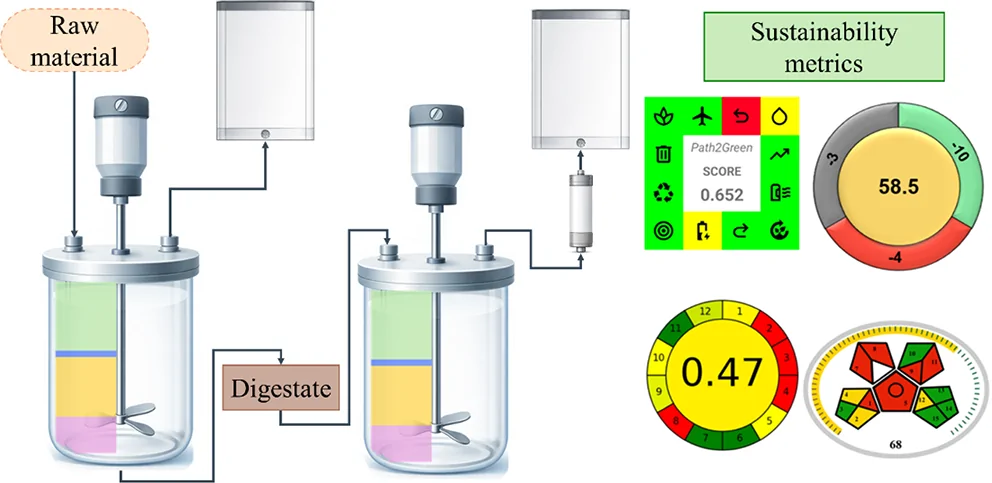

## 1 Introduction

The current energy and environmental crisis is forcing the world to shift from an energy matrix based primarily on fossil fuels to one composed of multiple renewable fuels.^1^ Renewable energy technologies currently available include diverse sources such as solar, wind, hydrogen (H_2_), hydropower, wave, and biomass, all of which are considered essential to the energy transition.^2^ The main challenges for the transition to a renewable energy matrix are (i) improving the efficiency of conventional energy conversion systems, (ii) integrating different renewable energy sources as most of them are intermittent, and (iii) providing adequate energy storage systems.^3^ There are also economic, infrastructure, and technological challenges to the adoption of renewable energy.^4^ For decision-makers to choose a truly viable path for energy transition, rapidly changing economic and technological factors must be continually evaluated.^5^

H_2_ is one of the most promising energy solutions, capable of meeting future energy needs with zero emissions,^6^ reducing environmental impacts, and creating new energy opportunities.^7^ The International Energy Agency (IEA) emphasizes the need for stronger policies to stimulate demand, fund demonstration projects, and expand infrastructure for low-emission hydrogen.^8^ H_2_ can be produced by various methods, such as hydrogen from fossil fuels (e.g., natural gas via steam methane reforming, SMR), from renewable sources (e.g., electrolysis of water using renewable energy), from nuclear energy, and from microbiological routes.^9^ SMR and coal gasification are cost-effective and well-established methods for H_2_ production, while the biological and light-driven methods are still under development.^10^

The dark fermentation of organic waste, residual biomass, and effluents is gaining attention as an economical and environmentally friendly method for producing H_2_.^11^ In this process, certain microorganisms, such as *Clostridium*, *Thermoanaerobacterium* spp. and *Enterobacter*, ferment carbohydrate-rich organic matter and convert it to H_2_, carbon dioxide (CO_2_) and volatile fatty acids (VFAs), such as acetic and butyric acid.^12^ Agricultural and agro-industrial by-products have the potential to produce_H2_. In addition, these materials are cheap, readily available, and biodegradable, making them even more attractive for revalorization.^13^ Some biomasses, especially lignocellulosic biomass, require pretreatment with acids, bases, or enzymes to break down lignin and release hemicellulose as fermentable sugars.^14^ Pretreatment methods improve H_2_ yield and can be applied to the substrate and/or the inoculum.^15^

Sequential reactor studies reported in the literature use the first reactor for H_2_ production and the second reactor for CH_4_ production.^16^ The second reactor can also be used for H_2_ production, usually using dark fermentation processes followed by photofermentation.^17^ In this configuration, the first reactor (dark fermentation) produces hydrogen from the conversion of glucose, forming organic acids such as acetic, butyric, and succinic acids, which serve as substrates for the second reactor.^18^ The photofermentation reactor uses the organic acids produced in the dark fermentation to produce more hydrogen, usually through the action of photosynthetic bacteria.^19^ This configuration is used to improve overall hydrogen production.

H_2_ can be used in fuel cells to generate electricity, heat, and water without emitting greenhouse gases, making it a sustainable and clean option.^20^ Major applications include fuel cell electric vehicles, power and heat generation for buildings and industry.^21^ It is also used in the production of ammonia for fertilizers and in oil refining processes in the chemical industry.^15^ It is essential for decarbonization because it can replace fossil fuels in sectors that are difficult to electrify (such as heavy-duty transportation and high-temperature industries).^22^

Energy production methods need to be evaluated to determine which offer the best opportunities to support the transformation of the global energy matrix. In this sense, the 12 Principles of Green Chemistry provide a methodological framework for developing and implementing chemical products and processes that are compatible with sustainability and environmental criteria.^23^ Several free tools enable sustainable evaluation of analytical and experimental procedures, such as Eco-Scale,^24^ Path2Green,^25^ AGREE,^26^ and GAPI.^27^

This study aims to produce hydrogen-rich biogas from apple and cashew pomace, two relevant residues in Brazil, to promote their valorization through anaerobic conversion. The proposed experiment will be carried out in two sequential reactors, with the first serving as a pretreatment step to improve the overall process performance. In addition, a simple and accessible gas purification strategy will be applied to improve biogas quality. Finally, the proposed process is evaluated using the Eco-Scale, Path2Green, AGREE, and GAPI sustainability metrics. This work presents dry anaerobic digestion in 4.3 liter reactors, demonstrating promising operational performance and favorable biogas production compared to values commonly reported in the literature. By integrating this sequential system with fuel cell electricity generation, the study demonstrates the feasibility of converting agro-industrial liabilities into clean energy. This systemic approach, using sustainability metrics, points to biogenic hydrogen as a strategic energy vector and confirms the validity of the circular bioeconomy proposal.

## 2 Methods

### 2.1 Raw Materials and Inoculum

Apple pomace (AP) and cashew pomace (CP) were collected in the city of Campinas, São Paulo State, Brazil. The by-products were dried in an oven (model 315-SE, brand Fanem, Brazil) at 60 °C for 72 h and then ground in a blender until a fine powder (∼1 mm) was obtained. The prepared substrates were packaged in plastic bags and stored at −4 °C until used in the experimental tests, in order to preserve their physical and chemical characteristics and to avoid microbial contamination. The AmBev brewery (Jaguariúna, SP, Brazil) provided the mesophilic inoculum used in the experiment, which was used without any pretreatment. The inoculum is a complex consortium of microorganisms coming from wastewater treatment plants, consisting mainly of bacteria and *Archaea*. The experimental system consisted of two sequential anaerobic reactors with different primary functions. The first reactor, referred to as the “hydrolysis reactor,” was primarily responsible for substrate degradation and solubilization, while the second reactor, referred to as the “dark fermentation reactor,” was associated with hydrogen production.

Raw materials, inoculum, and the operational performance of the system were analyzed according to the Standard Methods for the Examination of Water and Wastewater.^28^ The parameters evaluated included pH (Method 4500-H^+^ B), total solids (TS) (Method 2540 B), total fixed solids (TFS), total volatile solids (TVS) (Method 2540 E), and chemical oxygen demand (COD) (Method 5220 D). All analytical determinations were performed in triplicate to ensure reproducibility. However, these triplicates refer only to the analytical procedures and not to the independent biological reactor replicates. When concentrations exceeded the upper limit of quantitation (ULOQ), samples were diluted as necessary and results were corrected using the dilution factor. Table 1 shows the main parameters for the initial characterization of the substrates and inoculum used in the experiment.

**Table 1:** Characterization of the Raw Materials and Reactors Used in the Experiment, Including Physicochemical Parameters, Operating Conditions, and Hydrogen Production (Mean ± Standard Deviation)^a^ ^b^

				Reactors					
		Biomass		Hydrolysis			Dark fermentation		
Parameter	Inoculum	AP	CP	Initial	Final	Average	Initial	Final	Average
pH	4.04	-	-	6.8	4.62	5.49	7.5	7.02	7.1
Total solids (%)	20.11 ± 1.94	91.25 0.08	78.87 ± 2.7	28.04 ± 0.16	22.33 ± 0.31	23.31 ± 0.41	31.73 ± 0.13	19.82 ± 0.49	22.45 ± 0.45
Fixed solids (%)	2.66 ± 0.03	4.14 ± 1.92	1.64 ± 0.02	2.21 ± 0.07	7.39 ± 0.43	6.27 ± 0.51	1.95 ± 0.01	6.72 ± 0.45	6.22 ± 0.20
Volatile solids (%)	17.45 ± 1.22	87.11 ± 2.0	77.23 ± 2.72	25.83 ± 0.09	14.94 ± 0.59	17.05 ± 0.53	29.77 ± 0.14	13.10 ± 0.21	16.21 ± 0.43
Moisture (%)	79.89 ± 1.19	8.75 ± 0.08	21.13 ± 2.7	71.96 ± 0.16	77.67 ± 0.31	76.69 ± 0.41	68.27 ± 0.13	80.18 ± 0.49	77.39 ± 0.41
Chemical oxygen demand (g O_2_/L)	23.27 ± 4.9	80.38 ± 0.67	119.9 ± 0.9	144.87 ± 3.26	108.20 ± 1.17	135.75 ± 2.81	146.52 ± 1.30	65.30 ± 2.29	112.47 ± 3.82
Hydraulic retention time (days)				48.16					
Organic loading rate (g COD/L·day)				238.06					
Experimental hydrogen yield (m^3^/ton)				93.40					

a AP—apple pomace.

b CP—cashew pomace.

### 2.2 Experimental Configuration of Anaerobic Reactors

Figure 1 shows a flow diagram of the experimental setup used in this study. Two identical laboratory-scale anaerobic reactors with a total volume of 4.3 L were used and operated in semi-continuous mode for 39 days. Figure 1A shows how the reactors were fed with 60% of their total capacity (2.58 L), corresponding to the substrate, inoculum and water mixture, with the remaining 40% (1.72 L) reserved as headspace for the accumulation of biogas generated during the experiment. The composition of the substrate mixture was prepared and added to each reactor. The mixture consisted of 95 g cashew pomace (CP), 487 g apple pomace (AP, both ground and dry), 1508 g wet inoculum and 160 g water. Thermostatic baths (Tecnal, TE-2005, São Paulo, SP, Brazil) maintained the temperature at 36 °C to favor the growth and activity of the microbial consortium. Figure 1B shows a difference in the operation of the reactors: the hydrolysis reactor supplied the feed to the dark fermentation reactor, as explained in the following paragraphs.

**Figure 1:**
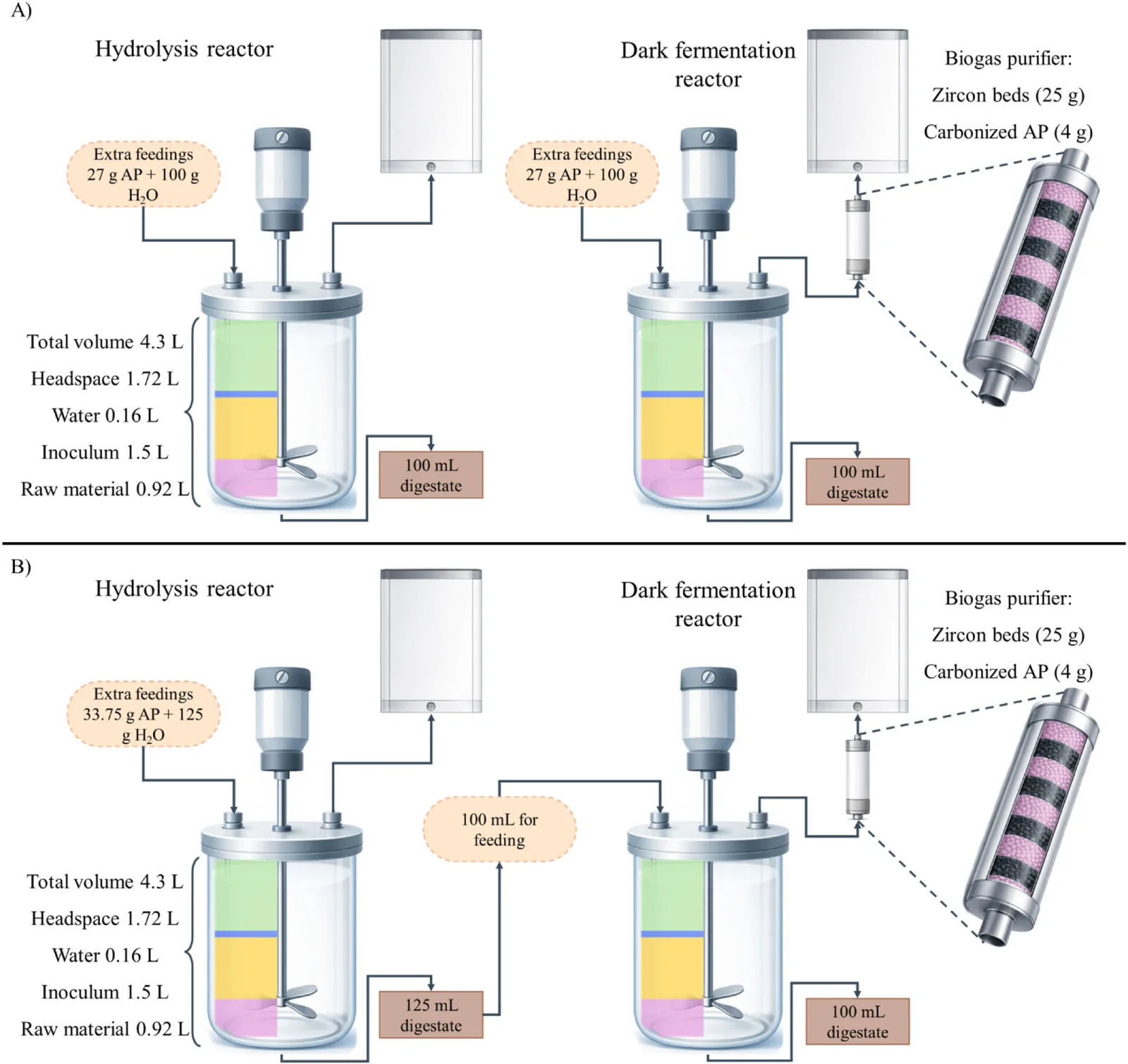
Schematic representation of the experimental setup for biomass pretreatment (hydrolysis) and dark fermentation. A) the initial phase of the experiment, when both reactors were operated identically, and B) the operating phase, corresponding to day 6, when the dark fermentation reactor was fed with digestate from the hydrolysis reactor. (Source: Created by the authors).

Figure 2 presents an experimental diagram that divides the study into two phases. Figure 2a shows the initial phase of the experiment in which the two reactors were operated identically. First, each reactor received the substrate mixture. Then, on the second and fourth days of the experiment, the reactors received supplemental feeding. These supplements consisted of 27 g AP and 100 mL water. Simultaneously, 100 mL of digestate was withdrawn.

**Figure 2:**
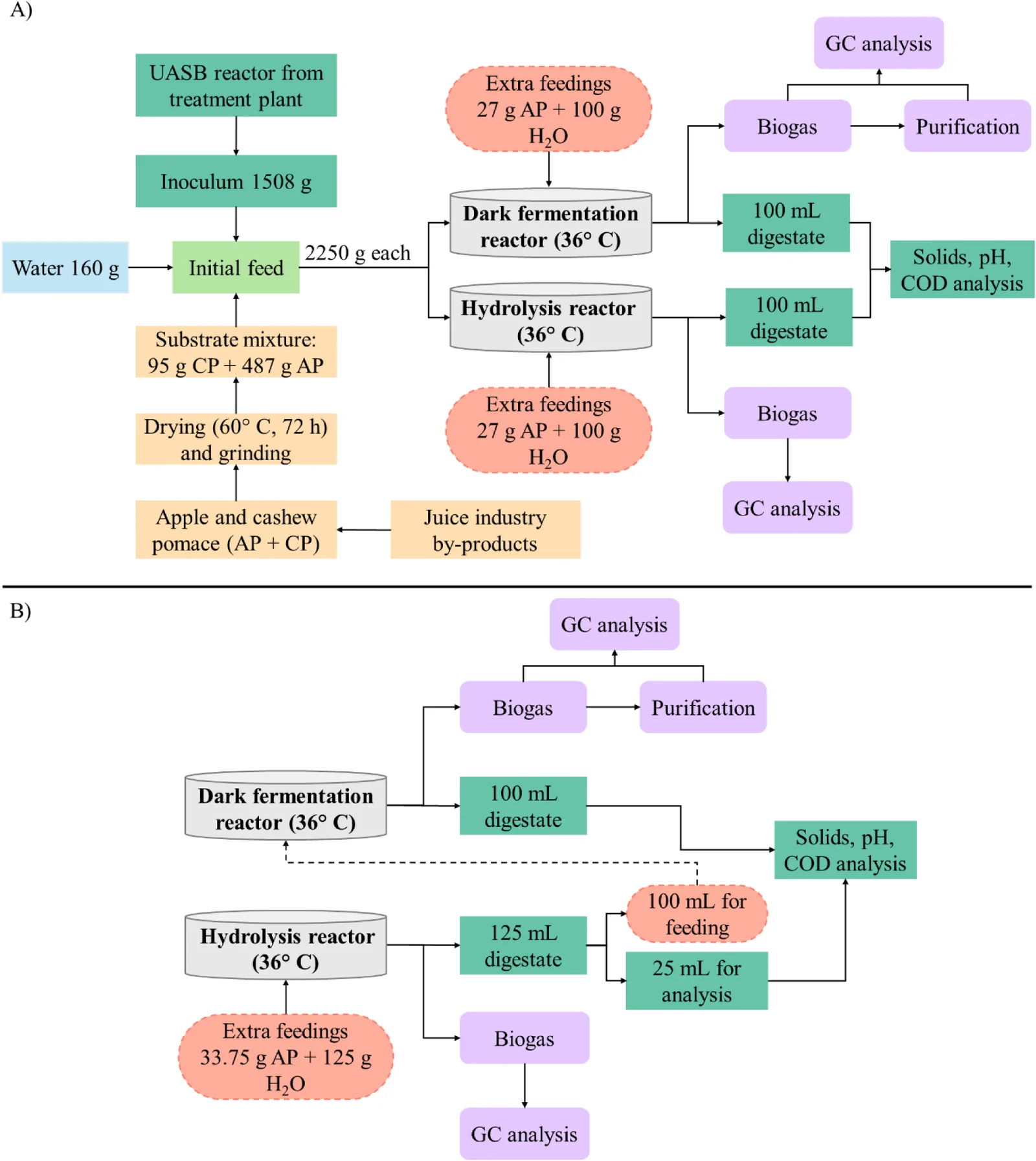
Flow chart of the experimental operation with two reactors (hydrolysis and dark fermentation) showing A) the initial phase of the experiment, when the two reactors were operated identically, and B) the operating phase, corresponding to day 6 onwards, when the dark fermentation reactor was fed with digestate from the hydrolysis reactor.

Figure 2b shows the operational phase, which corresponds to the sixth day. During this period, the hydrolysis reactor was fed with 33.75 g of AP and 125 mL of water, and 125 mL of digestate was removed. These 125 mL of digestate were divided into two streams: 25 mL for analysis and 100 mL to feed the dark fermentation reactor. The dark fermentation reactor was fed with 100 mL of digestate from the hydrolysis and the same volume (100 mL) of sample was withdrawn. This procedure was repeated three times a week until the end of the experiment. During the start-up and operational phases, the amount of feed added each day was equal to the amount of digestate removed, thus maintaining constant reactor working volumes.

During the experiment, the pH of the reactors was adjusted between 5.0 and 6.5 for the hydrolysis reactor and between 6.0 and 8.0 for the dark fermentation reactor by adding sodium hydroxide (NaOH, 6 mol/L). pH control is essential to maintain appropriate conditions for the initial hydrolysis of the substrate in the first reactor, which enables H_2_ production in the second reactor. Reactor contents were homogenized for 1 minute prior to sampling and after each system feed.

Process performance was evaluated by calculating the hydraulic retention time (HRT) (eq 1) and the organic load rate (OLR) (eq 2):^29^

1 $$HRT=\frac{V_{Reactor}}{Q_{Feed}}$$

2 $$OLR=\frac{Q_{Feed}\times S_{Feed}}{V_{Reactor}}$$

where HRT represents hydraulic retention time (days); OLR the applied organic loading rate (mg VS/L·day or, alternatively, g COD/L·day); Q_Feed_ feed flow rate (L/day); S_Feed_ the feed substrate concentration (g VS/L of feed or, alternatively, g COD/L of feed); and V_Reactor_ the reactor working volume (L).

Biogas was collected daily in Tedlar bags (Supelco Analytical, Darmstadt, Germany) connected to each reactor. Biogas was quantified using a syringe that aspirated the entire volume accumulated in each bag. The sum of the daily volumes represented the cumulative biogas production throughout the experiment. Unlike the hydrolysis reactor, the dark fermentation reactor had a biogas purification system using carbonized AP. The composition of the biogas was analyzed for both reactors. For the dark fermentation reactor, the composition was analyzed by collecting the sample directly from the reactor (i.e., before purification) and also from the Tedlar bag (purified).

The composition of the biogas was determined by gas chromatography using a Shimadzu® system (model GC-2014) equipped with a thermal conductivity detector (TCD). The separation of the components—hydrogen (H_2_), hydrogen sulfide (H_2_S), methane (CH_4_), and CO_2_—was performed in a Carboxen 1010 PLOT fused silica capillary column, 30 m × 0.53 mm. Nitrogen (N_2_) was used as the carrier gas, with a flow rate of 6 mL/min and a pressure of 5 bar. The injected biogas sample volume was 0.5 mL, and the injector and detector temperatures were maintained at 150 °C. The thermal program consisted of an isocratic at 75 °C held for 12 min. The components were quantified by normalizing the areas of the chromatographic peaks after calibration of TCD with standards for each gas, and the results were expressed as a percentages (%).

### 2.3 Carbonized Apple Pomace

Carbonized AP was prepared from dry, ground AP by carbonization under low O_2_ concentration (O_2_ depletion). According to the literature, the residual material of various raw materials already shows satisfactory pore formation when carbonized at 400 °C, so this temperature was chosen.^30^ First, the mass of AP was placed in a porcelain crucible and heated in a muffle furnace (model CE-800/S2, brand Cienlab) at 400 °C for 4 h.^30^ After cooling the carbonized material in the muffle, the resulting solid product was weighed and transferred to a mortar where it was pounded with a pestle until uniformly distributed particles were obtained. eq 3 was used to estimate the yield of carbonized material^31^

3 $$Carbonized\;material\;yield=\left(m_{carbonized}/m_{AP}\right)\times 100\%$$

Where *m_carbonized_* is the mass of carbonized material obtained after carbonization, and *m_AP_* is the mass of AP used to get the carbonized AP.

Next, 4 g of pulverized carbonized AP was placed in a metal cartridge (109 mm long and 32 mm internal diameter) with cotton filters at each end. Inside the cartridge, the material was arranged in alternating layers of 1 g of carbonized AP and 5 g of zirconia spheres (3 mm diameter), for a total of 25 g of zirconia spheres. The zirconia beads were used as an inert material to prevent the formation of preferential gas pathways through the charred material, thereby avoiding dead zones that could compromise the performance of the purifier. The cartridge was placed after the dark fermentation reactor and before the Tedlar bag for biogas collection.

The resulting carbonized material was examined by scanning electron microscopy (SEM) using a Hitachi TM-4000 Plus microscope (Tokyo, Japan) before and after use in biogas purification. Before SEM examination, the samples were fixed on double-sided carbon tape. Visualization was performed in BSE mode (10 kV).

Thermogravimetric analysis (TGA) and differential scanning calorimetry (DSC) were used to evaluate the thermal stability and decomposition behavior of the carbonized AP samples. Analyses were performed using a high-precision thermal analyzer (model: Jupiter STA 449 F5—NETZSCH). The mass of the sample used for the TGA evaluation was 10 mg. The sample was transferred to an aluminum crucible, and the atmosphere was kept inert with nitrogen gas (flow rate of 20 mL/min). Analyses were performed at temperatures ranging from 30 to 1,000 °C (heating rate of 10 °C/min).

### 2.4 Experimental Hydrogen Yield

The experimental yield of accumulated H_2_ was determined according to eq 4, corresponding to the parameter *EHY_added_* (m^3^ *H*_2_/ton biomass), respectively:

4 $$EHY_{added}\left(\frac{m^{3}H_{2}}{ton_{biomass}}\right)=\frac{V_{biogas}\times H_{2}}{{ton}_{biomass}}$$

Where, *V_biogas_* represents the accumulated volume of biogas produced during the experimental period (m^3^), and H_2_ corresponds to the percentage fraction of H_2_ in the biogas mixture; *ton_biomass_* corresponds to the total mass of biomass introduced into the reactor (ton), including the substrate at the initial and subsequent dosages.

### 2.5 Theoretical Bioenergy Recovery and Energy Consumption

The generation of electrical energy from the experimental production of biogas and H_2_ was evaluated for use in a fuel cell. The estimates were based on the assumption that the process would take place adjacent to the industrial plant where the residues are generated, and that the residues would then be subjected to hydrolysis and dark fermentation. First, the produced H_2_ was assumed to behave as an ideal gas. The volume of H_2_ was measured under laboratory conditions (25 °C, 1 atm). The molar amount of produced H_2_ was then calculated using the ideal gas law, and the resulting values were normalized to standard temperature and pressure (STP: 0 °C and 1 atm) to ensure accurate reporting and comparison with literature data. Finally, the mass yield was calculated. Knowing that the maximum electricity that could be obtained from H_2_ is equivalent to 237.13 kJ/mol of H_2_ (standard Gibbs free energy variation). Proton exchange membrane fuel cells (PEMFCs) typically have efficiencies of between 40% and 50%,^32^ thus an efficiency of 50% was considered in the present study. Equation 5 represents how the electricity production was calculated:^33^

5 $$Electricity=n_{hydrogen}\times {\Delta G}^{{}^{\circ}}\times \eta_{fuel\;cell}\times CF$$

where *n_hydrogen_* is the number of moles of H_2_ available for consumption, Δ*G*° is the standard Gibbs free energy variation, equal to −237.13 kJ/mol of H_2_; *η_fuel cell_* represents the fuel cell efficiency in converting H_2_ into electrical energy (50%), and CF is the conversion factor from kilojoules to kilowatt-hours (1 kWh = 3,600 kJ).

The energy consumption in each part of the AD process was estimated to understand the overall energy balance. The calculations included the consumption of the mixer, thermostatic bath, drying oven, and crusher. For the mixer, the energy consumption was calculated by multiplying the nominal power of the equipment by the total operating time, taking into account the daily operating time and the total number of operating days. For the thermostatic bath, the heat required to heat the water in the system was estimated using sensible heat, and the resulting value was converted from thermal energy to equivalent units of electrical energy. The minimum energy required for the drying stage was estimated based on two components: the sensible heat required to heat the material to the drying temperature and the latent heat required to evaporate the water in the biomass. For the grinding stage, the energy consumption was estimated by multiplying the nominal power of the equipment by the operating time required to reduce the particle size.

### 2.6 Green Metrics Analysis

The sustainability of the anaerobic digestion process of apple and cashew pomace was evaluated using the Eco-Scale, Path2Green, AGREE and GAPI metrics. These metrics allow the quantification and qualification of environmental impacts, covering principles related to energy consumption, process safety and waste management. This study was examined in comparison with other literature research on different anaerobic digestion methods, the samples, and the products obtained from biomass valorization. The first study explored the valorization of macaúba husks from biodiesel production using subcritical water hydrolysis pretreatment followed by anaerobic digestion, resulting in methane (CH_4_) production.^34^ The second study investigated the dry anaerobic digestion of apple pomace to produce methane (CH_4_) as part of a biorefinery approach for a circular bioeconomy.^29^ Each study employed different methods and samples, leading to distinct products and outcomes, which are summarized in Table 2 for comparison.

**Table 2:** Eco-Scale Assessment of the Anaerobic Digestion Processes Evaluated in This Study, Including the Applied Principles and the Corresponding Sustainability Scores Obtained for Each Experimental Condition

Reference	Yield (%)	Relative yield (%)	Method	Reagents	Technical/setup	Price	Safety	Temperature/time	Work and purification	Score
Apple and cashew pomace	40.70	71	hydrolysis pretreatment (first reactor) + anaerobic digestion (dark fermentation)	NaOH 6M	–4	0	–10	–3	–10	**58.50**
Study 1	57.00	100	Subcritical water hydrolysis pretreatment + anaerobic digestion	NaOH 6M	–7	0	–10	–3	–10	**70.00**
Study 2	20.26	35.34	Dry anaerobic digestion	NaOH 6M	–4	0	–10	–3	–10	**40.77**

Eco-Scale is an online software that assigns penalties based on number of principles, including yield, cost, safety, process time, temperature, preparation, and purification conditions eq 6 shows the method used to assign scores, where the penalties for each principle analyzed are subtracted from a base of 100. The sustainability of the method is rated based on the final score: an approach with a score above 75 is considered excellent, one above 50 is acceptable, and one below 50 is inadequate:^24^

6 Eco‐Scale=100−sumofindividualpenalties

Path2Green is an approach based on the 12 Principles of Green Chemistry and serves as a basis for analyzing and verifying the sustainability of extraction methods, providing an objective, measurable assessment of associated impacts while integrating the environmental, social and economic pillars of sustainability. This metric assigns a score and rating based on parameters such as biomass used, pretreatment applied, yield, energy consumption, product purification requirements, and waste management, facilitating detailed comparisons between methods and identifying more sustainable alternatives throughout the process. The score assigned to each method is represented by a value and an image, with color scales ranging from red (lowest score) to green (highest score).^25^

AGREE (Analytical AGREEness calculator) is a tool that applies the 12 Principles of Green Analytical Chemistry to identify and assign scores to the methods analyzed to assess environmental impact and sustainability. This easy-to-interpret tool translates complex parameters into a simple, intuitive index that allows direct comparisons between methods, identifies critical points, and guides improvements in analytical development. The calculator assigns a score between 0 and 1 based on parameters such as the degree of process automation, operator safety, and the types of reagents used. Each method receives a score that is displayed in numerical values and visual representations using a color scale from red to green, corresponding to the lowest and highest scores, respectively.^26^

The Green Analytical Procedure Index (GAPI) is a technique for analyzing analytical procedures based on their environmental impact, thereby inferring the environmental impact of the method. This tool evaluates analytical procedures in a more comprehensive, visual way, avoiding a focus on isolated steps and considering the entire procedure, identifying critical points in each method and encouraging continuous improvement of laboratory protocols. This metric generates a score and an image with five pentagrams that assess the sustainability of each stage of the methodology analyzed, assigning three colors: green for low impact, yellow for medium impact, and red for high impact. The parameters used are divided into three groups: those related to sample preparation, such as the type of sample preservation; those related to solvents and reagents; and those related to process instrumentation, such as the need to manage the waste generated.^27^

It is worth noting that although the Eco-Scale, Path2Green, AGREE, and GAPI metrics were developed to assess the sustainability of analytical chemistry extraction methods and processes, their application can be extended to other experimental contexts with appropriate adaptations. Anaerobic digestion is a process that involves biomass, solvents, energy consumption, and waste generation, and is directly related to all of the metrics mentioned. Thus, the use of these tools enabled a systematic, comparative assessment of the sustainability of the process, providing quantitative and qualitative parameters that help identify strengths and areas for improvement, even beyond the original scope of the tools.

## 3 Results and Discussion

### 3.1 Operational Parameters

In this study, the values calculated for the operating parameters were HRT of 48.16 days, OLR of 595.85 mg SV/L·day or 238.06 g COD/L·day (both based on the working volume of the reactor), and 93.40 m^3^ H_2_/ton biomass of EHY (at laboratory conditions 25 °C and 1 atm). These parameters are related to the composition of the substrate and may vary depending on microbial activity and operating temperature.^35,36^

Monitoring of key operating parameters such as pH, TS, VS, and COD provides fundamental insight into the biochemical transformations occurring in the two-stage biohydrogen production system using apple and cashew pomace. These variables reflect the balance between acid production and buffering capacity, the degree of substrate hydrolysis and solubilization, and the degree of organic matter conversion. The analysis of these parameters provided an overall assessment of the process performance and stability in each reactor, although it did not allow the identification of the metabolic pathways. In this sense, the first reactor is associated with an initial hydrolysis/acidification stage, followed by a hydrogen-producing dark fermentation.

#### 3.1.1 pH Control during Hydrolysis and Dark Fermentation

pH is one of the most critical operating parameters in anaerobic digestion because it directly affects the activity and community structure of microorganisms in mixed cultures and controls the dominant metabolic pathways, thereby determining biohydrogen production yields. The optimal initial and operating pH ranges depend on the characteristics of the inoculum and the type of organic substrate being treated.^37^ While most studies agree that anaerobic digestion can occur over a wide pH range of 5.50–8.50, even slight variations can significantly affect microbial metabolism, reaction kinetics, and consequently biogas production rates.^38^ In addition, the ideal pH range varies depending on the substrate being fed into the reactor, making it difficult to maintain the proper pH.^39^

Because hydrogen production is inherently accompanied by the formation of organic acids (primarily acetic, lactic, butyric, and propionic) the pH of the medium gradually decreases during fermentation. When the pH falls outside the optimal range, the accumulation of these acids inhibits hydrogenase activity and disrupts intracellular pH homeostasis, leading to metabolic stress and a shift toward non-hydrogen producing pathways. Conversely, excessively alkaline conditions disrupt microbial homeostasis and reduce acidogenic efficiency, thereby limiting the production of soluble substrates required for subsequent methanogenesis.^40,41^ Therefore, maintaining a specific optimal pH window, especially in systems using food and fruit processing wastes and in pH-phased two-stage configurations, is critical to achieving functional separation of microbial activities and the most efficient biohydrogen production.

The reactors were operated at different pH ranges. This separation not only optimizes the metabolic performance of each consortium, but also minimizes competition between microbial groups. In the hydrolysis reactor, maintaining an acidic to slightly acidic pH (approximately 4.5–6.5) favors the activity of hydrolytic and acidogenic bacteria, which break down complex organic matter derived from food industry residues into simpler compounds. Under these conditions, the hydrolysis of carbohydrates, proteins, and lipids is promoted, followed by acidogenesis, in which fermentative bacteria convert the resulting monomers into VFAs and alcohols. At this stage, acetogenesis begins, in which some of the VFAs are converted to acetate, hydrogen and CO_2_. The low pH selectively inhibits methanogenic microorganisms that would otherwise consume the produced hydrogen, ensuring that the biohydrogen yield remains high.^42^ In contrast, the second reactor (dark fermentation) is kept at a neutral to slightly alkaline pH (about 6.5–8.5). The microbial groups in the second reactor use the previously degraded organic matter to produce even more H_2_, completing its degradation. In the present study, methanogen suppression was inferred from indirect experimental evidence, including the low methane production observed throughout the process and the persistence of acidic pH conditions during reactor operation. Although necessary to confirm methanogen suppression, VFA profiling and microbial community analysis were not performed.

#### 3.1.2 Solids

At the beginning of the experiment, the hydrolysis reactor showed TS and VS values of about 28% and 26%, respectively (according to Table 1). A significant decrease was observed during the first 14 days of digestion, with TS values around 21% and VS values around 13%. This sharp initial reduction indicates intense microbial activity during early hydrolysis, when easily degradable polysaccharides and soluble fractions of apple and cashew pomace are rapidly converted to monomers and short-chain organic acids. Such behavior is typical of hydrolytic-acidogenic systems treating fruit-based substrates rich in pectin and sugars.^43^

The dark fermentation reactor showed a similar but more pronounced decline in solids. Initial TS values of 32% (Table 1) decreased to about 20% by day 14, while VS decreased from about 30% to about 13%. This larger decrease reflects the consumption of the solubilized compounds produced in the first step, which served as the main carbon source for the fermentative metabolism. In contrast to the hydrolysis reactor, which showed a stabilization after the initial decrease, the dark fermentation reactor maintained a gradual downward trend in solids until day 20–25, followed by a stabilization around 19%. This behavior is consistent with systems in which the second stage receives a continuous influx of soluble organic matter from hydrolysis, allowing for prolonged fermentative activity and higher substrate conversion efficiencies.^39^

#### 3.1.3 COD

The COD profiles show clear differences in the degradation dynamics between the hydrolysis and dark fermentation reactors. In the hydrolysis reactor, COD remained relatively stable during the first 2 weeks of operation, fluctuating around 148 g O_2_/L. This behavior indicates that although the particulate matter was hydrolyzed, as confirmed by the decrease in TS and VS, the solubilized compounds accumulated in the liquid phase and were not substantially consumed in this reactor. Similar patterns have been reported in the hydrolysis-acidogenesis of fruit and vegetable wastes, where rapid solubilization of pectin and sugar-rich fractions leads to an increase or stabilization of soluble COD in the first stage.^43^ After about day 20, the COD in the hydrolysis reactor showed a slightly decreasing trend, reaching values close to 110 g O_2_/L toward the end of the experiment. However, the COD removal in this first stage remained moderate compared to the changes observed in the second reactor, reinforcing its role primarily as a solubilization step rather than a conversion unit.

The dark fermentation reactor, on the other hand, showed a markedly different COD degradation pattern. COD values remained similar to those of the hydrolysis reactor for the first few days, but then declined sharply, reaching approximately 52 g O_2_/L by day 35. This pronounced reduction reflects the active consumption of soluble organic matter from the hydrolysis reactor, consistent with the onset of enhanced fermentative metabolism. Substantial COD removal during dark fermentation of fruit pomace and other carbohydrate-rich residues has been widely reported, as hydrogenogenic microorganisms rapidly metabolize soluble sugars and volatile fatty acids under favorable pH and substrate conditions. Therefore, the dark fermentation digester served as the primary COD removal step in the system, completing the degradation of the soluble organic matter produced during the hydrolysis stage.^44^

### 3.2 Biogas Production

Figure 3a and b show the SEM images obtained for the carbonized AP used for biogas purification before and after the test, and Figure 3c and 3d show the results of TGA and DSG analysis of the carbonized AP before and after use. Figure 3a shows that before use, the carbonized AP has pores of different sizes (macropores and mesopores) typical of carbonized plant biomass, as well as irregular, rough surfaces with voids. In contrast, after use (Figure 3b), the carbonized AP has fewer visible pores and appears less rough, indicating a morphological change during biogas purification. Residual biomass is inexpensive and promising for obtaining charcoal with high adsorption capacity.^45^ Pore development is influenced by inorganic impurities and the initial structure of the precursor material.^45^ The adsorption properties of charcoal depend on its surface area, porosity, and the functional groups present on its surface, and it is often used for the removal of toxic gases and in the treatment of water and wastewater.^46^ The effects of temperature (200–600 °C) and pyrolysis time on the production of wheat straw and lignosulfonate char were evaluated. The biochar produced at 400 °C and 600 °C had more cracks and holes on its surface, suggesting an increase in surface area that may favor more adsorption sites. Temperature was found to have a greater effect on biochar quality, while pyrolysis time did not have a significant effect.^47^

**Figure 3:**
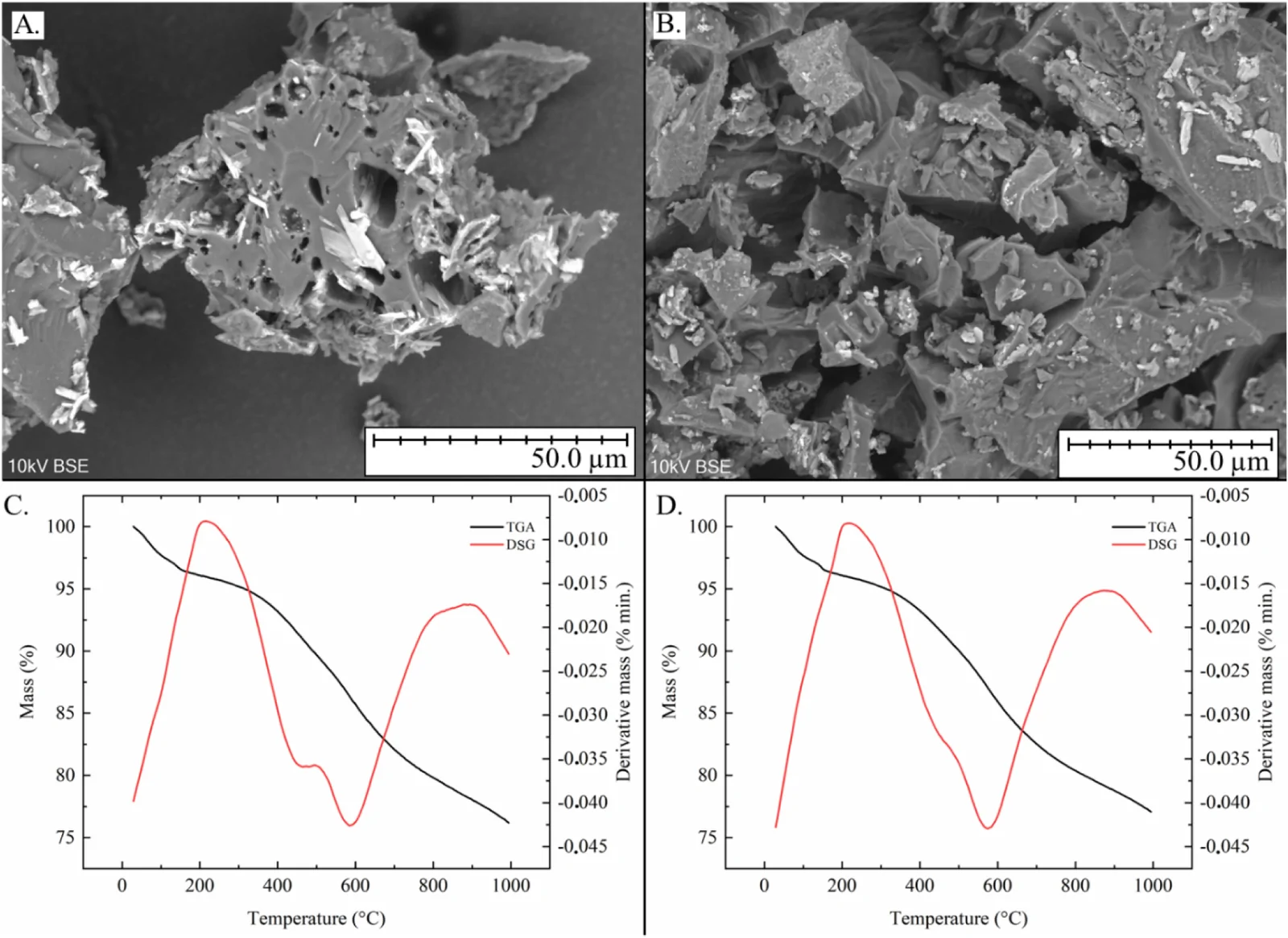
Scanning electron microscopy (SEM) images of carbonized apple pomace before (A) and after (B) application in biogas purification, obtained using backscattered electron mode (BSE, 10 kV). Thermogravimetric (TGA) and derivative thermogravimetric (DTG) profiles of the carbonized material before (C) and after (D) use in the purification process are also presented.

Thermogravimetric analysis is often used to predict the thermochemical behavior of materials by measuring changes in mass as a function of temperature or time under controlled atmospheric conditions. While DTG records the rate of change (derivative) of mass loss as a function of temperature or time, showing peaks that correspond to the maximum decomposition rates of the material’s components^48^ Figure 3c and 3d show a slight initial loss of mass, indicating loss of moisture and light volatiles (from 0 to 200 °C).^49^ In the intermediate range (200 to 600 degrees), there is a significant loss of mass corresponding to the degradation of hemicellulose, cellulose, and lignin or unstable carbon.^49^ In the range of 450 to 600 °C, a difference is observed between unused charcoal, which shows a plateau, and used charcoal, which does not, suggesting a probable transformation of functional groups during adsorption. Finally, above 600 °C, the DTG increases again, which may indicate the oxidation of more stable carbons or the decomposition of inorganic salts/impurities (such as carbonates and sulfates).^49^

Although biogas purification was not the central objective of the research, the preliminary results provide a basis for developing alternative routes to conventional technologies. The low-cost strategy outlined is not intended to replace established systems immediately, but rather to pave the way for future investigations that can optimize adsorption and provide deeper quantitative assessments.

The hydrogen volumes presented in this section were measured directly under laboratory conditions (25 °C and 1 atm). During the experiment, an average biogas production of 1,663.85 mL/day (or 1.66 × 10^–3^ m^3^/day) was observed in the hydrolysis reactor (totaling 64,890 mL or 6.49 × 10^–2^ m^3^). In comparison, 1,048.59 mL/day (or 1.05 × 10^–3^ m^3^/day) was observed for dark fermentation (totalizing 40,895 mL or 4.09 × 10^–2^ m^3^ of biogas). Therefore, greater biogas production was observed in the hydrolysis reactor. Figure 4 shows the results for daily and accumulated biogas production for the hydrolysis reactor (a) and dark fermentation (b). In the hydrolysis reactor, a peak in biogas production was observed on day 1, followed by fluctuations from days 2 to 16, and a significant drop from day 17, with an average production of only 722.48 mL/day (or 7.22 × 10^–4^ m^3^/day) between days 17 and 39. During dark fermentation, biogas production increased from days 1 to 5, with a peak on day 5 (3,236 mL or 3.24 × 10^–3^m^3^). From days 6 to 36, production ranged from 315 mL/day (or 3.15 × 10^–4^ m^3^/day, day 15) to 2,250 mL/day (or 2.25 × 10^–3^ m^3^/day, day 11), with an average of 726.97 mL/day (or 7.27 × 10^–4^ m^3^/day). After day 36, biogas production increased and H_2_ accounted for approximately 70% of the gas. In addition, a decrease in COD was observed throughout the experiment. Total solids in the dark fermentation reactor, on the other hand, were below 22% from the second week of the experiment and remained stable from day 14 to 39. These results suggest improved dark fermentation performance and greater conversion of organic matter under stable operating conditions.

**Figure 4:**
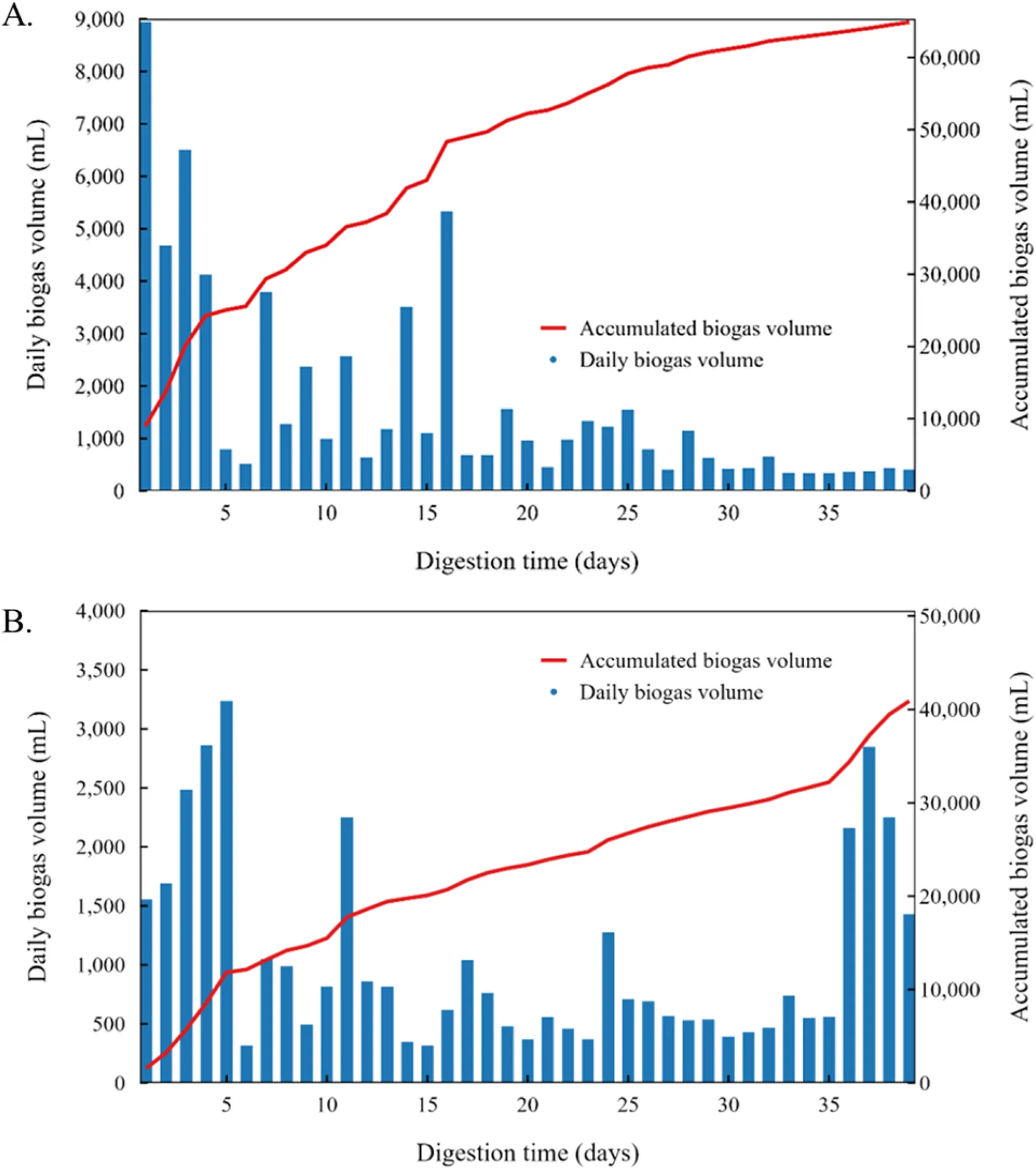
Daily and accumulated biogas production during the 39 days of experiment for A) hydrolysis reactor and B) dark fermentation reactor.

One study compared pretreatment with strong and weak acids under both pressurized and non-pressurized conditions to increase the bioavailability of biomass to microorganisms. The best pretreatment identified was pressurized sulfuric acid (1.2 atm and 121 °C), which achieved the highest total sugar concentration (23.71 g/L); however, the study did not perform dark fermentation.^50^ In another study, acidic (HCl) and alkaline (NaOH) pretreatments were tested to improve H_2_ production during dark fermentation of grass, with the best yield of 72.21 mL/g (or m^3^/ton) of dry biomass obtained with 4% HCl pretreatment.^51^ Considering the production of both reactors in the present study, a yield of 93.40 m^3^ H_2_/ton dry biomass was achieved. Dark fermentation alone does not produce enough hydrogen to achieve economic viability; therefore, the integration of other technologies with dark fermentation is being investigated to maximize hydrogen recovery from organic substrates.^37^

Figure 5a shows the daily biogas composition for both reactors and for the purification system. In contrast, Figure 5B shows the weighted average of the total biogas composition for each reactor and for the purified biogas. It can be observed that both reactors produced hydrogen at the beginning of the experiment (until day 7 and 11, respectively). After that, hydrogen production decreased, but increased again in the dark fermentation after day 25. The H_2_ production peaks were 77.48% for hydrolysis (day 1), 76.45% for dark fermentation (day 3), and 86.47% for purification (day 39). The biogas production under laboratory conditions results show higher production in the first reactor (64,890 and 40,895 mL or 6.49 × 10^–2^ and 4.09 × 10^–2^ m^3^ of biogas), however the weighted composition in terms of H_2_ was higher for the dark fermentation (Figure 4b). This behavior was expected for the configuration of the hydrolysis reactor working as a pre-treatment step, indicating that the first reactor was effective in providing an initial breakdown of the feedstock, converting carbohydrates to simpler sugars, proteins to amino acids, and lipids to simple fatty acids, consisting of more accessible materials for the dark fermentation bacteria to produce hydrogen.^37^

**Figure 5:**
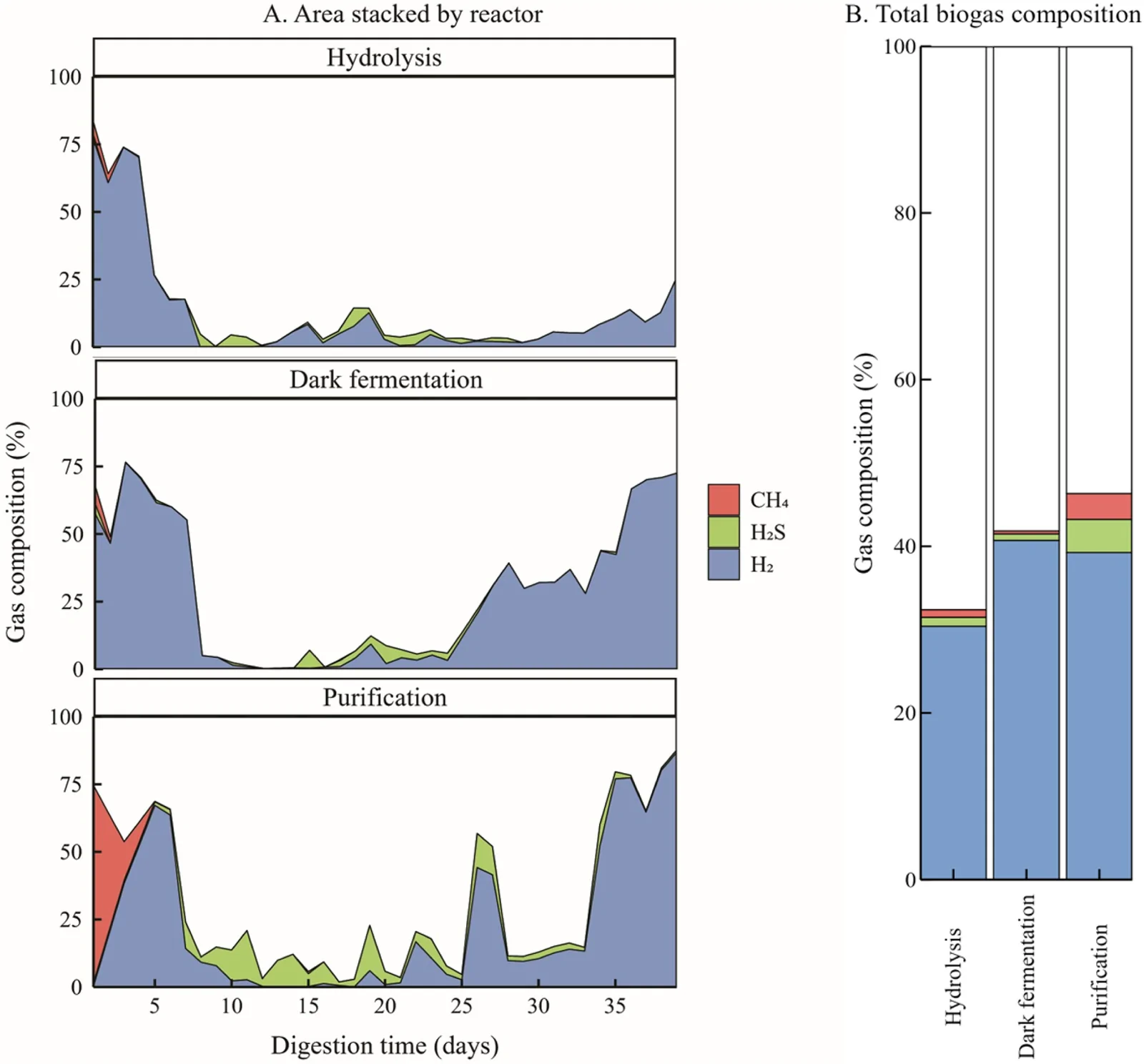
Biogas composition (H_2_, CO_2_, CH_4_, and H_2_S) for hydrolysis, dark fermentation, and the purification system (A) and weighted average composition in each case, calculated based on the respective biogas production volumes (B).

In terms of absolute H_2_ production, the volume produced in hydrolysis was 26,536.77 mL (2.65 × 10^–2^ m^3^) and 16,644.27 mL (1.66 × 10^–2^ m^3^) in dark fermentation (under laboratory conditions). Therefore, the proposed integrated system (two sequential reactors) allowed a more efficient consumption of organic matter and a greater absolute production of biogas and H_2_ (43,181.04 mL of H_2_ or 4.32 × 10^–2^ m^3^). The EHY obtained in the present study was 93.40 m^3^ H_2_/ton biomass or 107.36 m^3^ H_2_/ton VS_added_ (40.82% H_2_ considering the integrated system). The literature indicates results of H_2_ production by dark fermentation: 85 m^3^/ton VS_added_ for potato waste,^52^ 72 m^3^ H_2_/ton VS_added_ for kitchen waste.^53^ This indicates that the use of sequential reactors promotes greater H_2_ recovery, as expected and as described in dark photofermentation processes.^18^ According to the literature, the percentage of H_2_ in biogas varies with different operating conditions and substrates during dark fermentation.^54^ For example, kitchen waste produced 46% H_2_ in biogas (inclined plug flow reactor, continuous operation),^53^ while municipal solid organic waste produced 55% H_2_ (CSTR, semi-continuous).^55^ The hydrogen yields reported in the literature vary widely, reflecting how sensitive this process is to reactor design, mode of operation, and data normalization. A prime example is the high yield achieved by Náthia-Neves et al. (2018)^56^ using a semi-continuous thermophilic CSTR to process a highly biodegradable blend of food waste and vinasse. This high-temperature, fluid system is far different from the sequential batch design used in the present study, which operates under lower moisture conditions. Conversely, complex solid matrices present their own structural bottlenecks, such as the batch digestion of untreated brewer’s spent grains by Sganzerla et al. (2023)^57^ resulted in much lower yields due to lignocellulosic recalcitrance. Explicitly tracking these differences in residence time, temperature, and feed regimes prevents misleading comparisons and allows to demonstrate how the sequential configuration can provide an optimized balance between operational simplicity and energy efficiency.

For the purification cartridge coupled to the dark fermentation reactor for biogas purification, the weighted average biogas composition was 30.41% H_2_ and 67.60% CO_2_ for hydrolysis; 40.70% H_2_ and 58.16% CO_2_ for dark fermentation; and 39.25% H_2_ and 53.67% CO_2_ for purification (Figure 5b). Comparing the dark fermentation composition with the purified biogas composition, the result indicates that the purification system, although fluctuating during the experiment, was still effective overall in retaining CO_2_ from the biogas. After purification, the H_2_ percentage decreased, while the H_2_S and CH_4_ percentages increased due to CO_2_ retention. This behavior occurs because the removal of CO_2_ changes the calculation basis for the gas mixture, even though the absolute amounts of the remaining gases are not changed. In addition, it should be noted that the purification system implemented was not pressurized, which could have yielded better results. Also, the purification system presented in this study is a preliminary proof of concept, as adsorption capacity, breakthrough curves and repeatability have not been demonstrated.

### 3.3 First-Order Energy Balance Estimation

For the energy generation analysis, the hydrogen volumes were normalized to STP (0 °C, 1 atm) to ensure standardized calculations. One way to convert H_2_ to energy is through a fuel cell. In a proton exchange membrane fuel cell, H_2_ enters the cell on the anode side and is oxidized, splitting into 2 protons (H^+^) and 2 electrons (e^–^) with the help of a catalyst. The protons (H^+^) are allowed to pass through the membrane and go to the cathode side, but the electrons (e^–^) are not. Electrons are forced to pass through an external circuit, creating an electric current. On the cathode side, the protons that have crossed the membrane, the electrons from the external circuit, and the O_2_ from the air combine to form 2 molecules of H_2_O, and heat is released from the system.^58^

Figure 6 is a schematic representation of a biorefinery processing apples and cashews, producing AP and CP as by-products. For the first-order energy production calculations, the experimental results of H_2_ production presented in this study were considered. These results were used to plan the treatment of 1 ton of by-products (following the proportions established in Figure 1). The number of moles of H_2_ resulting from the integrated treatment of this amount of material was calculated (4,167.04 mol of H_2_/ton of by-products). Using a fuel cell with 50% efficiency, 137.24 kWh of electricity is generated.

**Figure 6:**
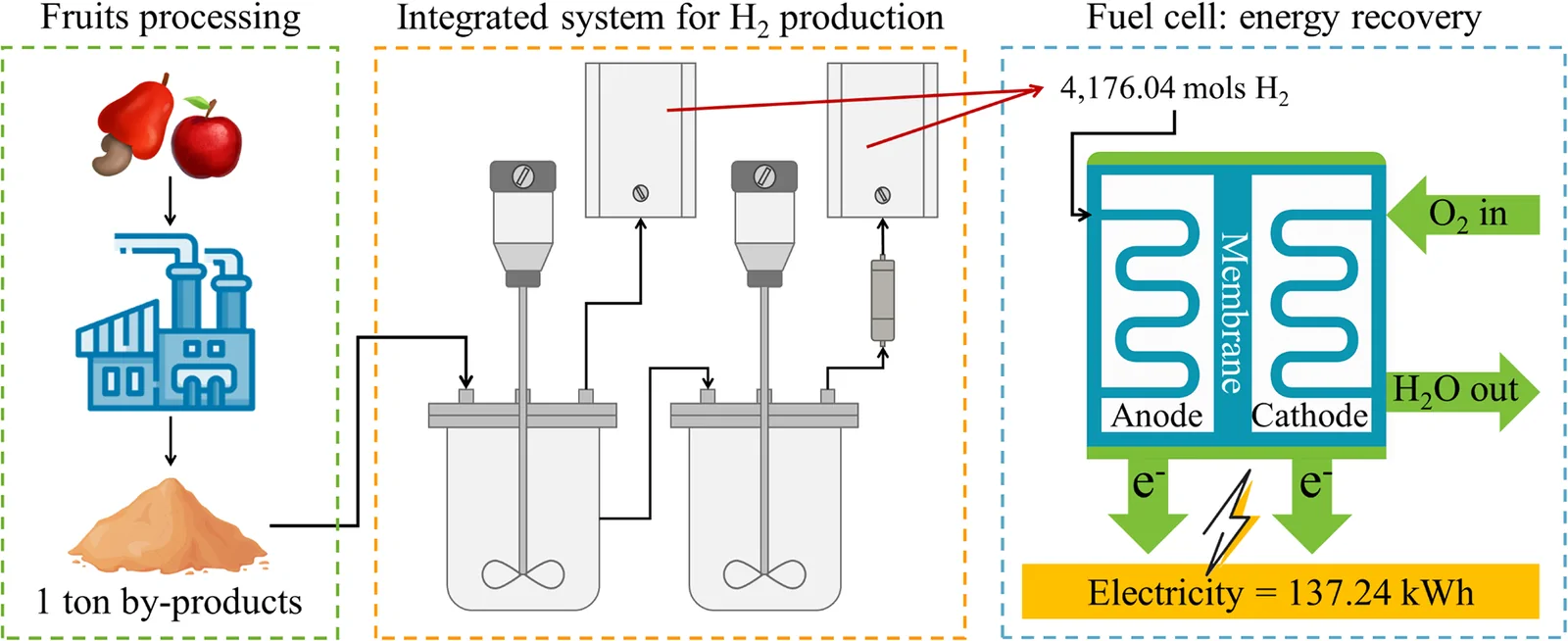
Schematic representation of industrial processing of apple and cashew apple in a biorefinery, emphasizing the generation of solid by-products (apple pomace, AP and cashew pomace, CP). The by-products undergo an integrated treatment for hydrogen (H_2_) production, which is subsequently utilized in a fuel cell to generate electricity. (Source: Created by the authors).

Regarding the simplified estimated energy consumption for the laboratory scale, the results were 78 Wh for the mixer, 109.21 Wh for the thermostatic bath, 1279.02 Wh for the oven used to dry the substrate, and 125 Wh for the grinder, for a total of 1,591.23 Wh. From these calculations it is possible to see the percentage contribution of each activity to energy consumption, which is 4.90% for the mixer, 6.86% for the thermostatic bath, 80.38% for drying and 7.86% for the grinder. The drying of the substrate in the oven accounted for the highest energy consumption. If the drying stage were omitted, which is plausible in industrial applications where by-products can be processed wet, the energy consumption would be reduced to about 312.21 Wh, resulting in a consumption of 137.8 kWh/ton of biomass. With an estimated energy production of 137 kWh/ton of biomass, the process energy balance would be close to equilibrium. It is also worth noting that industrial-scale equipment tends to be more efficient, which could have a positive impact on the energy balance.

In a previous study by the author’s research group, a CSTR reactor was operated in semi-continuous mode for 40 days in dry-type anaerobic digestion to produce CH_4_. Dried AP was used as feedstock at 36 °C with pH adjusted to 6–8. The reactor operation showed frequent pH drops, indicating that the material may be suitable for H_2_ production. The energy recovery results obtained in the study were 1.9 kWh of electricity and 8.6 MJ of heat per ton of AP in a combined heat and power (CHP) system (motor efficiency of 40% for electrical energy and 50% for thermal energy).^29^ Although preliminary estimates of first-order power recovery suggest that dark fermentation has greater potential, a definitive comparison is currently limited by the need for further studies.

Another study found in the literature evaluated the following routes for apple pomace management: composting, anaerobic digestion, and dark fermentation, followed by anaerobic digestion.^59^ Composting is applied on a small scale, producing compost that can be used as a biofertilizer, but it has a higher environmental impact due to emissions. Anaerobic digestion produces biogas containing CH_4_, reduces direct greenhouse gas emissions, and is a widely studied and viable technology on an industrial scale; however, it requires pH control, consumes chemicals, and is less efficient at using organic matter than dark fermentation.^59^ Finally, dark fermentation followed by anaerobic digestion produces biohydrogen and biogas separately. This increases the efficiency of using organic matter and stabilizes the digestate. However, this process requires neutralization between steps and presents technical challenges related to stability. According to the study, the two-step process (dark fermentation followed by anaerobic digestion) allows for the greatest energy recovery and organic matter degradation.^59^ The treatment of cashew pomace by dark fermentation has also been studied. Acid hydrolysis of cashew pomace yielded up to 72% H_2_ in the produced biogas, indicating its suitability for energy applications.^60^

### 3.4 Sustainability Analysis

The AGREE, Path2Green, GAPI, and Eco-Scale metrics are all based on fundamental principles for evaluating the sustainability of analytical processes. These principles include parameters such as reagent consumption, yield, process time, purification, and safety. Despite their different methodologies and scoring scales, all of these metrics are based on the principles of green chemistry, which enables integrated and comparative analysis. These sustainability metrics were adapted specifically for this framework, and the findings should be interpreted with caution as they reflect a contextual comparison with existing literature.

Penalty points and final scores for each parameter attributed to the proposed study and the others evaluated by the Eco-Scale metric are presented in Table 2. During the Eco-Scale evaluation, yield was assessed in relation to methane production. Regarding the “technical configuration” parameter, the option that best fit the evaluated processes was selected from the provided options. For this study and the others, “instruments for controlled addition of chemicals” and “glove box” were selected since a controlled reagent (NaOH) was added in all processes and gloves were required for operator safety during the process. For Study 2, “pressurized equipment (>1 atm)” was selected because this process included a hydrolysis step at pressures greater than 1 atm prior to AD. For the “temperature/time” parameter, “heating, >1 h” was selected for all processes since the AD process occurs at temperatures of approximately 36 °C. For the “processing and purification” parameter, “classical chromatography” was the only option selected for all processes, as this analysis is necessary for determining the composition of the produced biogas.

The proposed work received a score of 58.5, which is considered “acceptable” according to the metric’s classification. The other evaluated studies received similar scores. Compared to Study 1, the presented project stood out with respect to the “technical configuration” parameter because it did not require high-pressure equipment, thereby reducing energy consumption. Additionally, the developed method produced a high yield, particularly compared to Study 2.

The AGREE metric assigned a score from 0 to 1 based on the 12 principles of Green Analytical Chemistry (GAC) for the developed work and the evaluated studies (Table 3). Regarding parameter 1, all processes were classified as “offline” since none included an automated pretreatment step. For parameter 2, the amount of the sample used in the process was considered. Regarding Principle 3, all processes were considered “offline” since none were automated. However, the process could be automated by connecting the anaerobic digester to the gas chromatograph. For parameter 4, the steps performed during anaerobic digestion (AD) between the initial feeding and subsequent feedings were considered. For principle 5, all processes were considered manual because they are batch processes. They are also considered miniaturized because the introduced samples are ground, meaning the remaining digestate will also be ground. All processes received the maximum score for parameter 6 because none of them used derivatization agents. Since digestate can be used as a biofertilizer, under principle 7, it was considered that no waste was generated for all processes. For parameter 8, it was assumed for all processes that the “number of analytes determined in a single run” was 1 and the “number of analytes determined per hour” was 0 because nothing could be determined in one hour. It took at least 24 h to collect a sample and an additional 39 or 40 days to obtain the results (Studies 1 and 2). Principle 9 evaluates the energy consumed during the process. Among the options, the gas chromatograph (GC) was selected because it is the equipment used to analyze the composition of the biogas produced. In this study, approximately 0.000398 kWh/g was used. Thus, for the total amount of sample introduced into the reactor (1805.25 g), 0.72 kWh was consumed. In Study 1, approximately 0.01408 kWh/g was used. Thus, for the total amount of sample introduced into the reactor (76.36 g), 1.08 kWh was consumed. In Study 2, 0.000398 kWh/g was used. In the total amount of sample introduced into the reactor (1734.18 g), 0.69 kWh was used. Of the options provided in Principle 10, “some reagents are bio-based” was selected for all processes since NaOH (which is not of biological origin) and inoculum were used in all processes. For comparison purposes, inoculum was considered a bio-based reagent since it is a necessary material (microorganism) for carrying out the process. All processes received the same score for parameter 11 since no toxic reagents were used. Regarding Principle 12, “explosive” and “highly flammable” were selected for all processes because biogas generation at the end of AD may present these risks.

**Table 3:** AGREE Parameters and Scores Adopted for the Anaerobic Digestion Processes Studied

Parameters	Apple and cashew pomace	Study 1	Study 2
Parameter 1—Direct analytical techniques should be applied to avoid sample treatment	0.48	0.48	0.48
Parameter 2—Minimal sample size and minimal number of samples are goals	–2.50	–1.17	–2.48
Parameter 3—*In situ* measurements should be performed	0.00	0.00	0.00
Parameter 4—Integration of analytical processes and operations saves energy and reduces the use of reagents	0.00	0.00	0.00
Parameter 5—Automated and miniaturized methods should be selected	0.50	0.50	0.50
Parameter 6—Derivatization should be avoided	1.00	1.00	1.00
Parameter 7—Generation of a large volume of analytical waste should be avoided and proper management of analytical waste should be provided	1.00	1.00	1.00
Parameter 8—Multianalyte or multiparameter methods are preferred versus methods using one analyte at a time	0.00	0.00	0.00
Parameter 9—The use of energy should be minimized	0.50	0.50	0.50
Parameter 10—Reagents obtained from renewable source should be preferred	0.50	0.50	0.50
Parameter 11—Toxic reagents should be eliminated or replaced	1.00	1.00	1.00
Parameter 12—The safety of the operator should be increased	0.60	0.60	0.60
Score	0.47	0.45	0.47

The proposed study received a score similar to that of Study 2 (0.47). Compared to Study 1, the main difference in the applied method was energy consumption. Since Study 1 used subcritical hydrolysis as a pretreatment step, it had higher energy consumption than the present study, resulting in a penalty under this principle.

The anaerobic digestion of apple and cashew pomace was evaluated using the Path2Green metric. Scores were assigned based on established principles, and the results were compared with those of other analyzed studies (see Table 4). For parameter 1, all processes received the same score since they all used food industry waste as the raw material. Principle 2 considers biomass transport. In the present study, the sample traveled 5 km from CEASA Campinas to UNICAMP. In Study 1, the sample traveled 10.3 km from the Campinas Agronomic Institute to UNICAMP. In Study 2, the sample traveled 820 km from the city of Videira to UNICAMP. Principle 3 considers pretreatment implementation. For the present study, a biological pretreatment was applied due to the use of inoculum and because one reactor was used to feed the other. Study 2 applied a physical pretreatment due to the use of hydrolysis with subcritical water. Pretreatment was not considered for Study 3. Parameter 4 evaluates solvent use. The use of NaOH was analyzed for all processes. For the present study, the “problematic solvent” option was considered due to the large quantity of NaOH used in the process (672 g). The same approach was taken in Study 2, which used 1,795 mL of NaOH. However, in Study 1, due to the low amount of NaOH used (126 mL), the “recommended solvent” option was considered. According to Principle 5, all processes were considered semicontinuous due to the feeding method applied. Regarding Principle 6, an additional purification step is required for all processes because the percentage of methane in the biogas is not ideal for application. For parameter 7, full utilization of the biomass was considered for all processes since what remains in the reactor at the end of the process (digestate) can be used as a biofertilizer. For parameter 8, the option “combining two post-treatments based on recommended solvents” was selected for all processes because an additional purification step is required for all processes to produce biogas with a higher percentage of methane. For the present study and Study 2, Principle 9 considered the option “Low-energy extraction technique using non-renewable energy” because both use less than 1 kWh of energy. For Study 1, which used more than 1 kWh of energy, the option “High-energy extraction technique using non-renewable energy” was considered. Regarding Principle 10, the application of this concept to all processes was considered. Since it falls within the energy sector, it has the potential to be used as a renewable energy source to support processes in other fields. For Parameter 11, the option “Non-virgin raw materials were used” was selected since waste was used as a raw material in all processes. For Principle 12, 0% waste generation was considered since the residue remaining in the reactor at the end of the process (digestate) can be used as a biofertilizer.

**Table 4:** Path2Green Parameters and Scores Adopted for the Anaerobic Digestion Processes Studied

Parameters	Apple and cashew pomace	Study 1	Study 2
Parameter 1—Biomass: select biomass that is naturally sourced or requires minimal resource usage for production	+1.00	+1.00	+1.00
Parameter 2—Transport: preserving biomass integrity while minimizing transport’s environmental impact	5 km	10 km	820 km
Parameter 3—Pretreatment: optimization for pre-treatment avoidance and cost-effective techniques	–0.30	–0.20	+1.00
Parameter 4—Solvents: minimize solvent usage, prioritizing those of biological origin, biodegradable and nontoxic	0.00	+1.00	0.00
Parameter 5—Scaling: ensure reproducibility and a continuous extraction flow	+0.50	+0.50	+0.50
Parameter 6—Purification: final application dictates the extent of purification	+0.50	+0.50	+0.50
Parameter 7—Yield: maximize the utilization and valorization of the biomass	+1.00	+1.00	+1.00
Parameter 8—Posttreatment: functionalization of natural products post-extraction to maximize their benefits	+0.50	+0.50	+0.50
Parameter 9—Energy: prioritize using clean energy sources and high-efficiency extraction techniques	0.00	–1.00	0.00
Parameter 10—Application: ensure safety for applications in several domains	+1.00	+1.00	+1.00
Parameter 11—Repurposing: trace strategies to perform closed loop extraction systems, preferably using non-virgin materials	+1.00	+1.00	+1.00
Parameter 12—Waste management: refine waste reduction and ensure effective waste management	0%	0%	0%
Score	0.652	0.659	0.597

As shown in the AGREE metric, the Path2Green score revealed that the difference between the developed method and Study 1 was related to energy consumption. Compared to Study 2, the current method stood out in the transportation parameter: the raw material used in Study 2 required transportation of approximately 820 km. According to this principle, raw materials must travel short distances between their sources and points of application to maintain their integrity and reduce environmental impact.

Table 5 shows the principles evaluated and their corresponding aspects as they relate to the present study and other works analyzed by the GAPI metric. The same considerations applied to Principle 1 as to AGREE Principle 1. For Parameter 2, only physical preservation was considered because all samples were stored under refrigeration prior to application. For Principle 3, the need for specialized sample transport was considered, but none of the procedures required it. For Parameter 4, normal storage conditions were considered for all processes. For Principle 5, the need for a simple sample preparation procedure was considered for all processes. Parameter 6 was considered “not applicable” for all cases. For Principle 7, the option “Non-green solvents/reagents used” was selected for all studies because they all used NaOH. Parameter 8 assesses the need for additional treatments. The same considerations as those in Path2Green Principle 6 were applied to this parameter, thereby selecting the option “advanced treatments.” Parameter 9 assesses the amount of reagent used. The option selected was based on the quantities used, as described in Path2Green Principle 4. For Principle 10, since there was no option to use nontoxic reagents, the mildest option, “slightly toxic,” was chosen for all processes, with a risk score ranging from 0 to 1. For Principle 11, the strictest option was chosen for all processes due to the explosion and flammability risks associated with biogas. For Principle 12, the score was applied using the same considerations described in AGREE Principle 9. For parameter 13, it was assumed that the process was hermetically sealed, as the reactors are sealed to prevent leaks. For Principle 14, the same considerations as Path2Green Principle 12 were applied, selecting the option “<1 mL (1 g)”. For parameter 15, the same considerations as Path2Green Principle 12 were applied to all processes. For comparison purposes, the “recycling” option was selected since the digestate can be reused as a biofertilizer. For Principle 16, “yes” was selected for all processes since they all allow for the quantification of biogas produced via GC.

**Table 5:** GAPI Parameters and Scores Adopted for the Anaerobic Digestion Processes Studied

Parameters	Apple and cashew pomace	Study 1	Study 2
Sample preparation			
Parameter 1—Collection	Offline	Offline	Offline
Parameter 2—Preservation	Chemical or physical	Chemical or physical	Chemical or physical
Parameter 3—Transport	None	None	None
Parameter 4—Storage	Under normal conditions	Under normal conditions	Under normal conditions
Parameter 5—Type of method	Extration required	Extration required	Extration required
Parameter 6—Scale of extraction	Not applicable	Not applicable	Not applicable
Parameter 7—Solvents/reagents	Nongreen solventes/reagentes used	Nongreen solventes/reagentes used	Nongreen solventes/reagentes used
Parameter 8—Additional treatment	Advanced treatments	Advanced treatments	Advanced treatments
Reagents and solvents			
Parameter 9—Amount	>100 mL (>100g)	>100 mL (>100g)	>100 mL (>100g)
Parameter 10—Health hazard	Slightly toxic, slightly irritant; NFPA health hazard score = 0 or 1	Slightly toxic, slightly irritant; NFPA health hazard score = 0 or 1	Slightly toxic, slightly irritant; NFPA health hazard score = 0 or 1
Parameter 11—Safety hazard	Highest NFPA flammability or instability score of 4	Highest NFPA flammability or instability score of 4	Highest NFPA flammability or instability score of 4
Instrumentation			
Parameter 12—Energy	≤1.5 kWh per sample	≤1.5 kWh per sample	≤1.5 kWh per sample
Parameter 13—Occupational hazard	Hermetic sealing of analytical process	Hermetic sealing of analytical process	Hermetic sealing of analytical process
Parameter 14—Waste	<1 mL (1 g)	<1 mL (1 g)	<1 mL (1 g)
Parameter 15—Waste treatment	Recycling	Recycling	Recycling
Quantification	Yes	Yes	Yes
Score	68	68	68

Figure 7 shows the final score and classification (green, yellow, or red) for each method and parameter, as indicated by the pentagrams. The present study and other studies received the same score, which highlights that none of the methods use toxic solvents.

**Figure 7:**
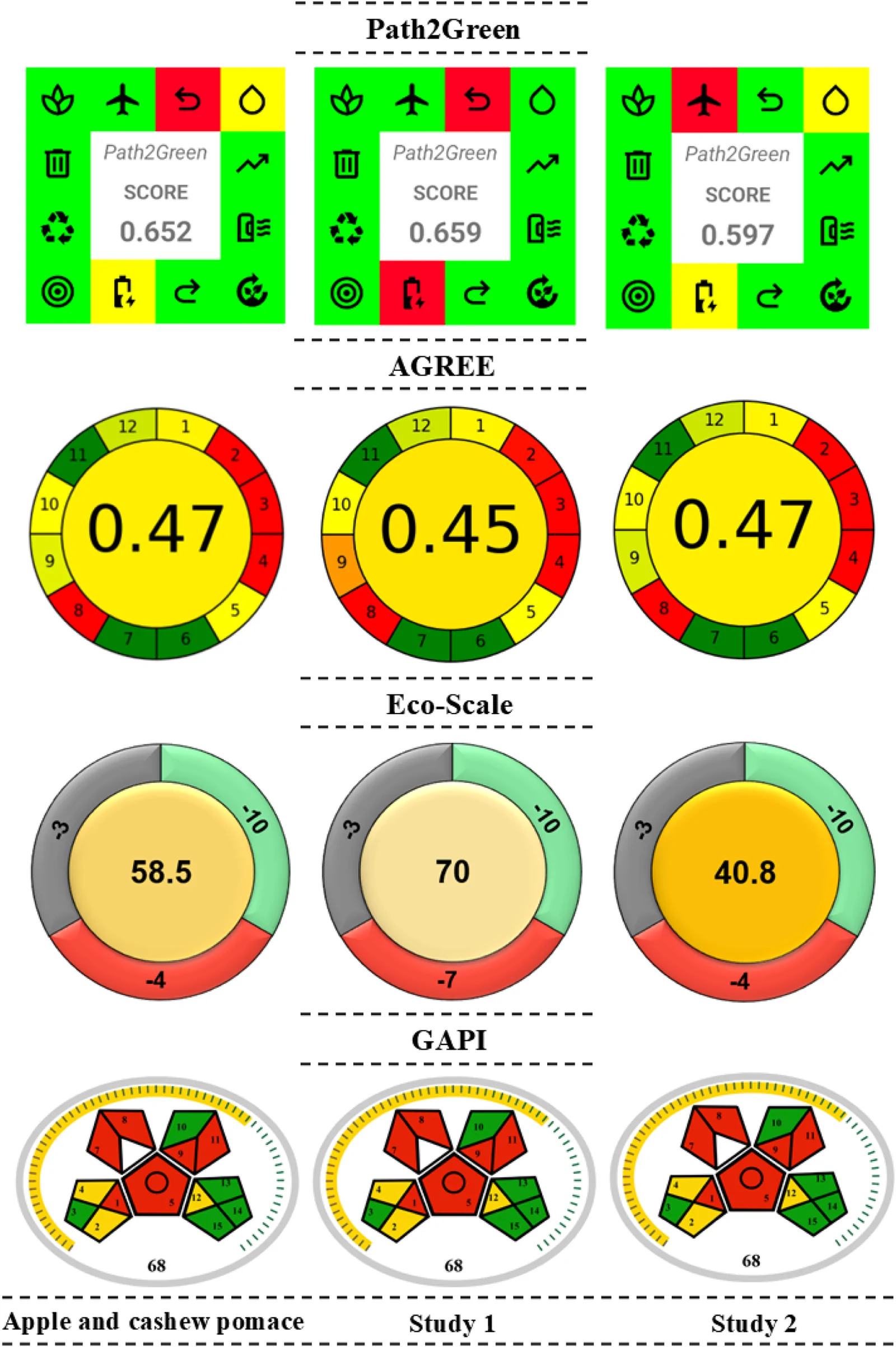
Comparison of the sustainability metric results obtained in this study with those reported in similar studies available in the literature, including Path2Green, AGREE, Eco-Scale, and GAPI assessments. (Source: Created by the authors).

Each sustainability metric has unique strengths and limitations that affect its usefulness in evaluating the anaerobic digestion process. This is evident from the different scores obtained for each metric. The Eco-Scale provides a direct, quantitative analysis based on penalty points, enabling quick comparisons between processes. However, its simplified structure may overlook broader environmental impacts. Path2Green is a multicriteria tool that addresses different operational aspects; however, its objective is to evaluate extraction methods, as is the case with the Eco-Scale. This can considerably influence the assignment of scores. GAPI and AGREE are metrics that address analytical processes in general, broadening their application and reducing the likelihood of deviations in the applied score. However, due to the complexity of some parameters, their application may be limited. Therefore, using all metrics together is essential to obtaining a more robust and reliable result.

Based on the applied tools, it can be concluded that the anaerobic digestion process of apple and cashew pomace aligns with the principles of green chemistry and demonstrates sustainability potential. In addition to the previously discussed parameters, the performance of the study is supported by the utilization of waste as a raw material, the absence of toxic solvents during the process, and the management of generated by-products, yielding a digestate with potential applications, such as in fertilizer production.^61^ However, environmental sustainability can be enhanced by improving certain parameters, such as reducing solvent volumes and implementing automated processes.

These sustainability assessment tools were originally designed for analytical processes. While these analyses were designed to evaluate the “sustainability check” in analytical methods, they also provide a new way to compare different approaches based on environmental impact and efficiency. In the present study, applying these tools enabled to compare different bioenergy production strategies and highlight aspects such as resource consumption, waste generation, and environmental impact. While adapting these analyses to biological processes may present limitations, using them contributes to an initial sustainability assessment by providing information that can be used to identify areas for improvement and guide future investigations.

## 4 Conclusions

This study investigated the production of biohydrogen from apple and cashew pomace using a two-stage anaerobic process. Under laboratory condition, the process yielded 93.40 m^3^ H_2_ per ton of biomass, accompanied by organic matter conversion and biogas production. The evaluation using green chemistry metrics suggested the potential sustainability of the process under the studied conditions. The integrated system of two anaerobic reactors appeared to facilitate the functional separation of microbial activities, potentially contributing to the conversion of organic matter into hydrogen.

An acceptable total volume of biogas was produced under laboratory conditions: 64,890 mL (or 6.45 × 10^–2^ m^3^) in the hydrolysis reactor and 40,895 mL (or 4.09 × 10^–2^ m^3^) in the dark fermentation reactor. Preliminary observations under the studied parameters suggest that the hydrolysis reactor primarily facilitated initial organic matter breakdown as a pretreatment step, while hydrogen production was tentatively driven by the dark fermentation reactor. Under the tested conditions, integrating the reactors seemed to enhance the efficiency of biogas and H_2_ production. The process demonstrated a decrease in total and volatile solids during the 39-day operation period, indicating organic matter conversion. The hydrolysis reactor appeared to facilitate the initial solubilization of organic compounds, while the dark fermentation reactor likely continued the degradation process, leading to organic matter consumption and subsequent biogas production.

Based on this preliminary setup, estimated hydrogen yields reached 4,167.04 mol H_2_/ton biomass, representing a potential generation of 137.24 kWh of electricity in an idealized 50% efficient fuel cell (calculated under STP). Based on the applied green chemistry metrics, the process demonstrated favorable preliminary indicators of sustainability due to waste utilization and the absence of toxic solvents, though extensive optimization is still needed to confirm its practical viability. A detailed quantitative evaluation of CO_2_ adsorption capacity, biochar breakthrough behavior, and hydraulic retention time (HRT) was not performed at this stage. Furthermore, the mechanistic interpretation was based primarily on indirect indicators (pH, solids, COD, and gas composition), as volatile fatty acid (VFA) profiles and microbial community analyses were not conducted. Consequently, the carbonized apple pomace purification cartridge, as well as the energy and sustainability assessments, remain early-stage proof-of-concept evaluations. Future studies employing synthetic biogas under controlled conditions are necessary to validate system performance and optimize the overall process. Overall, the findings concerning purification effectiveness, process feasibility, and broader sustainability remain preliminary and context-dependent, providing a foundation for future scaled-up investigations.
